# Mapping the gut microbiota-diabetic peripheral neuropathy research landscape: a bibliometric analysis of emerging trends and translational frontiers

**DOI:** 10.3389/fendo.2026.1815280

**Published:** 2026-05-13

**Authors:** Yuda Fei, Peng Mao, Bifa Fan

**Affiliations:** 1 Department of Infection Control, Peking Union Medical College Hospital, Chinese Academy of Medical Sciences & Peking Union Medical College, Beijing, China; 2 Graduate School, Beijing University of Chinese Medicine, Beijing, China; 3 Department of Pain Medicine, China-Japan Friendship Hospital, Beijing, China

**Keywords:** bibliometrics, CiteSpace, diabetic peripheral neuropathy, fecal microbiota transplantation, gut microbiota, gut-brain axis, Mendelian randomization, research trends

## Abstract

**Background:**

Diabetic peripheral neuropathy (DPN) constitutes the most prevalent chronic complication of diabetes mellitus, affecting approximately 50% of patients throughout their disease course. Accumulating evidence indicates that gut microbiota (GM) dysbiosis plays a pivotal role in DPN pathogenesis via the gut-brain axis. However, a comprehensive bibliometric analysis delineating the intellectual landscape and evolutionary trajectory of this rapidly advancing research domain remains absent.

**Methods:**

Publications pertaining to gut microbiota and DPN were systematically retrieved from the Web of Science, PubMed, and Scopus databases (spanning 2010–2025). CiteSpace 6.4.R1 was employed to perform co-occurrence, clustering, timeline, burst detection, and co-citation analyzes, thereby visualizing the field’s intellectual structure and developmental trends.

**Results:**

A total of 133 publications met the inclusion criteria, exhibiting exponential growth after 2018 with an average annual increase of 35.7%. Keyword analysis identified core research clusters centered on GM, neuropathic pain, the gut-brain axis, and therapeutic interventions such as fecal microbiota transplantation (FMT), traditional Chinese medicine, and causal inference methodologies like Mendelian randomization. Burst detection analysis revealed a temporal shift from foundational concepts (e.g., “oxidative stress”, “inflammation”) toward interventional strategies (e.g., “fecal microbiota transplantation”, “traditional Chinese medicine”) and causal inference approaches (e.g., “Mendelian randomization”). Co-citation analysis highlighted seminal contributions, including clinical trials demonstrating FMT efficacy and animal studies elucidating the role of microbial metabolites such as butyrate.

**Conclusion:**

This study presents the inaugural bibliometric analysis of the GM-DPN research field. The domain is transitioning from establishing associative links to elucidating causal mechanisms and evaluating targeted interventions. Keyword trend analysis underscores a convergence toward a multi-mechanistic gut-brain axis model. Future research priorities derived from the literature encompass clinical translation, multi-omics integration, and personalized therapeutic strategies.

## 1 Introduction

Diabetic peripheral neuropathy (DPN) represents the most frequent chronic complication of diabetes, impacting approximately 50% of patients and resulting in pain, sensory deficits, and diminished quality of life (1). Conventional management strategies, predominantly centered on glycemic control, demonstrate limited efficacy, highlighting an urgent need for novel therapeutic targets (2).

Over the past decade, the gut microbiota (GM) has emerged as a critical modulator of host metabolism and neurological function. The gut-brain axis—a bidirectional communication network involving neural, endocrine, and immune pathways—provides a conceptual framework for understanding how gut dysbiosis may contribute to DPN pathogenesis (3). Investigations in animal models and human subjects have implicated microbial metabolites, including short-chain fatty acids (SCFAs) and bile acids, in processes such as neuroinflammation and nerve injury (4, 5). Clinical trials have further explored interventional approaches, including probiotics, fecal microbiota transplantation (FMT), and electroacupuncture (EA) (6–9).

Despite growing research, no study has systematically mapped the intellectual structure, evolution, and emerging trends of this interdisciplinary field using quantitative methods. Bibliometric analysis offers a robust approach to visualize research landscapes and identify knowledge gaps (10).

Therefore, this study employs CiteSpace to conduct the first bibliometric analysis of publications on gut microbiota and DPN from 2010 to 2025. Our objectives are to delineate growth trajectories, decode thematic evolution, identify research hotspots and frontiers, and uncover foundational studies—providing a roadmap for future research.

## 2 Materials and methods

### 2.1 Data sources and search strategy

A systematic literature review was performed on January 15, 2025, utilizing three major bibliographic databases: Web of Science Core Collection (WoSCC), PubMed, and Scopus. The specific search strategies for each database were formulated as follows:

#### 2.1.1 WoSCC

(‘TS=((“gut microbiota” OR “gut microbiome” OR “intestinal flora” OR “gastrointestinal microbiome”)) AND (TS=((“diabetic peripheral neuropathy” OR “diabetic neuropathy” OR “diabetic polyneuropathy”))’.

PubMed:‘(“Gastrointestinal Microbiome”[Mesh] OR “gut microbiota”[tiab] OR “gut microbiome”[tiab] OR “intestinal microbiota”[tiab] OR “intestinal microbiome”[tiab] OR “gut flora”[tiab] OR “intestinal flora”[tiab] OR “gastrointestinal microbiome”[tiab]) AND (“Diabetic Neuropathies”[Mesh] OR “diabetic peripheral neuropathy”[tiab] OR “diabetic neuropathy”[tiab] OR “diabetic polyneuropathy”[tiab] OR “DPN”[tiab])’.

#### 2.1.2 Scopus

‘TITLE-ABS-KEY [(“gut microbiota” OR “gut microbiome” OR “intestinal microbiota” OR “intestinal microbiome” OR “gut flora” OR “intestinal flora” OR “gastrointestinal microbiome” OR “gastrointestinal flora”)] AND TITLE-ABS-KEY [(“diabetic peripheral neuropathy” OR “diabetic neuropathy” OR “diabetic polyneuropathy” OR “DPN”)]’.

The search was limited to publications in the English language. Eligible document types included original research articles and review articles. Duplicate records were initially identified and removed using EndNote X9 software (Clarivate Analytics), followed by a manual verification step to ensure accuracy.

### 2.2 Literature screening and study selection

**Figure 1:**
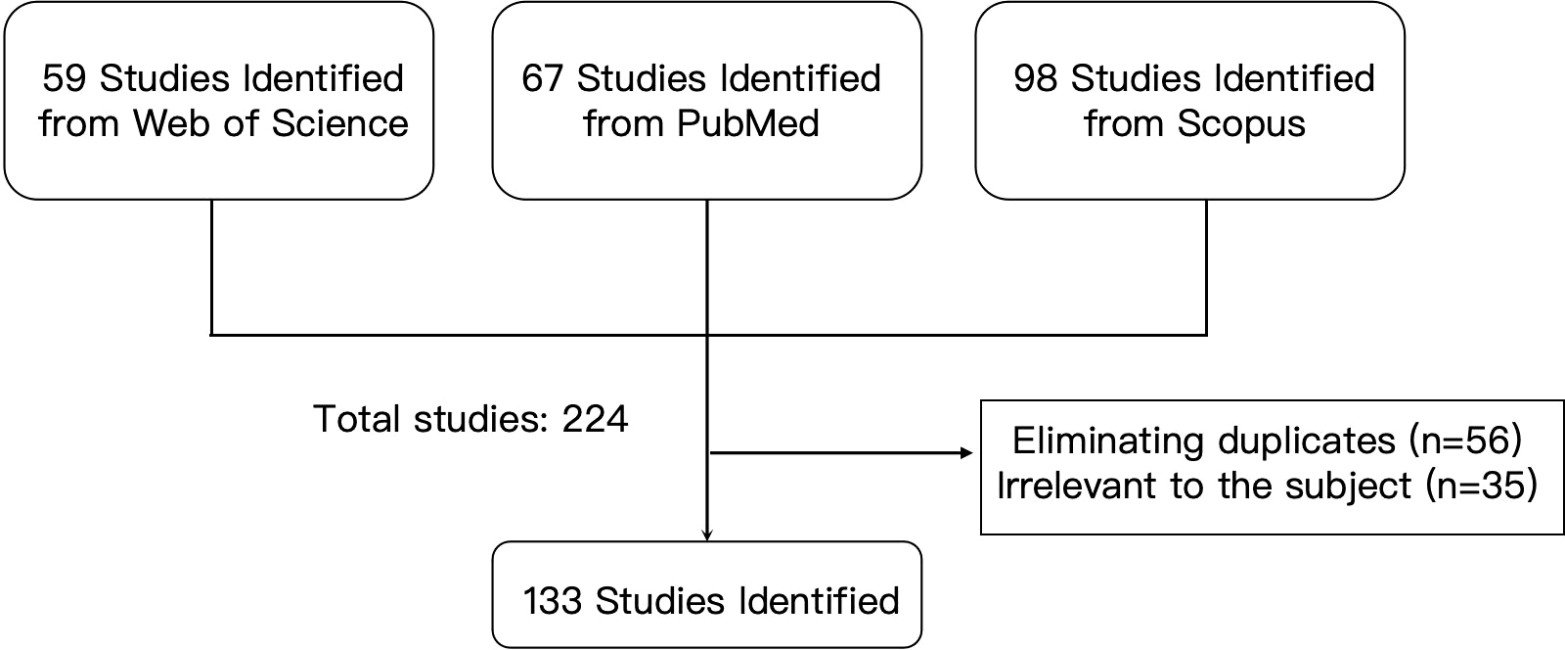
Flowchart of the literature retrieval and selection process for studies on gut microbiota and diabetic peripheral neuropathy.

The initial database searches retrieved 59 records from WoSCC, 67 from PubMed, and 98 from Scopus, resulting in a total of 224 publications. Following the removal of 56 duplicate records via EndNote X9 and subsequent manual verification, 168 unique records remained for screening. The screening process adhered to the PRISMA 2020 guidelines, and a corresponding flow diagram (**Figure 1**) illustrates the selection procedure to ensure transparency.

Titles and abstracts of the 168 records were independently screened by two investigators (Y.F. and P.M.) against the predefined eligibility criteria. Any discrepancies in screening decisions were adjudicated through consensus discussion or, when necessary, by consultation with a third investigator (B.F.). At this stage, 35 records were excluded primarily because they did not specifically investigate the association between gut microbiota and DPN (e.g., studies focusing on other diabetic complications such as retinopathy or nephropathy without neuropathy, or research on neuropathic pain in non-diabetic contexts). Consequently, 133 publications fulfilled all inclusion criteria and were incorporated into the subsequent bibliometric analysis (**Figure 1**). No age-based inclusion or exclusion criteria were applied, as this study analyzes publication trends rather than individual participant data.

The inclusion criteria were: (1) original research or review articles explicitly focusing on the relationship between gut microbiota (GM) and diabetic peripheral neuropathy (DPN) or its painful variant (DPNP); (2) articles published in the English language; (3) both preclinical (utilizing animal models) and clinical studies were eligible. Exclusion criteria encompassed: editorials, letters to the editor, conference proceedings, abstracts, and corrigenda; studies on diabetic complications without specific neural involvement; and studies investigating neuropathic pain in non-diabetic populations.

### 2.3 Data analysis and visualization

Prior to conducting the analysis, synonym merging and data cleaning were performed using a custom thesaurus file in CiteSpace. The thesaurus was constructed based on a preliminary analysis of high-frequency terms and established through consensus among the three authors. For instance, terms including “gut microbiota”, “gut microbiome”, “intestinal flora”, and “gastrointestinal microbiome” were merged into the unified term “gut microbiota”; similarly, “diabetic peripheral neuropathy”, “diabetic neuropathy”, and “diabetic polyneuropathy” were consolidated under “diabetic peripheral neuropathy”. This procedure was implemented to minimize node fragmentation and enhance the accuracy of subsequent clustering analyzes. While the process of synonym merging introduces a degree of subjectivity, a sensitivity check was performed: conducting the analysis without merging yielded qualitatively similar cluster structures but resulted in a lower modularity score (Q = 0.72 without merging versus Q = 0.84 with merging). This finding confirms that the implemented merging strategy improved conceptual clarity without distorting the core structural findings of the analysis.

All analyzes were conducted using CiteSpace (version 6.4.R1, 64-bit). The time span was configured from January 2010 to December 2025, with a one-year per slice interval. For node selection, the g-index was employed with a scaling factor k=25. This parameter value represents a balance between network comprehensiveness and selectivity; a higher k value would incorporate more low-frequency keywords, potentially increasing noise, while a lower k value might exclude potentially relevant emerging terms. The selection of k=25 aligns with commonly applied defaults in bibliometric studies utilizing CiteSpace (10). A sensitivity analysis employing k=20 and k=30 demonstrated stable core cluster structures, thereby confirming the robustness of this parameter choice. The Pathfinder and Pruning Sliced Networks algorithms were selected for network pruning to reduce structural complexity while preserving the most significant connections.

The following network analyzes and visualizations were generated:

#### 2.3.1 Keyword co-occurrence and clustering

The node type was specified as “Keyword”. The resulting network was clustered using the log-likelihood ratio (LLR) algorithm to identify major research themes.

#### 2.3.2 Keyword burst detection

Kleinberg’s algorithm was applied to detect keywords exhibiting a sharp, temporally constrained increase in citation frequency, indicative of emerging research frontiers.

#### 2.3.3 Document co-citation analysis

The node type was set to “Cited Reference”. This analysis identifies frequently co-cited publications, which typically represent foundational knowledge or pivotal studies within the research domain.

#### 2.3.4 Timeline and timezone views

These visualizations were derived from the clustered keyword network to depict the temporal evolution and inter-thematic relationships among research topics.

#### 2.3.5 Publication trend

Annual publication counts and cumulative totals were graphically represented.

The quality and homogeneity of the identified clusters were assessed using Modularity (Q) and Mean Silhouette scores. All exported network visualizations were subsequently refined using graphic design software to enhance clarity, visual coherence, and to ensure they met standard publication requirements.

It is important to note that no formal quality assessment of individual publications (e.g., evaluation of risk of bias or study quality scoring) was performed, as this bibliometric analysis focuses on aggregate publication patterns and knowledge structure rather than on evidence synthesis. Consequently, meta-analytic statistical methods, such as random-effects models, were not applied.

## 3 Results

### 3.1 Annual publication trends

**Figure 2:**
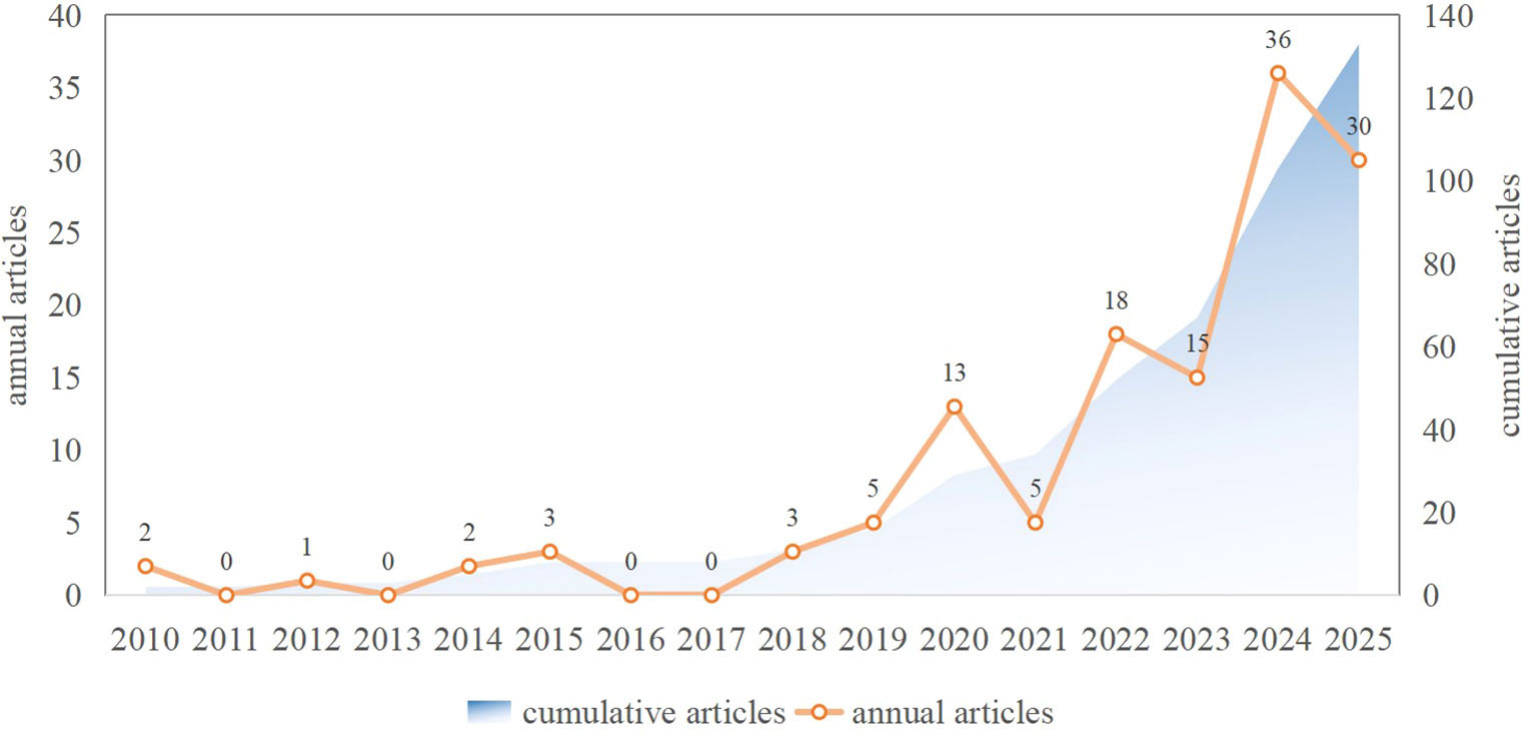
Annual publication trend of research on gut microbiota and diabetic peripheral neuropathy (2010–2025). The orange line denotes the number of publications per year; the blue area represents the cumulative number of publications.

A total of 133 publications pertaining to gut microbiota and diabetic peripheral neuropathy (DPN) were identified spanning the period from 2010 to 2025. The annual publication counts and cumulative trend are depicted in **Figure 2**. The field exhibited a latent phase from 2010 to 2017, characterized by fewer than 10 publications per year. A pronounced inflection point emerged around 2018, followed by a sharp increase in annual output. Peak publication numbers were recorded in 2023 (36 articles) and 2024 (30 articles). The cumulative publication count demonstrates a marked acceleration, particularly post-2020, reflecting the escalating research interest in the gut microbiota-DPN (GM-DPN) interface. A slight decline was noted in 2024 (30 articles) relative to 2023 (36 articles), which may be partly attributable to delays in database indexing, given that the search was conducted in early 2025. On average, 8.9 publications were published annually, with the highest annual output occurring in 2023.

### 3.2 Knowledge structure: keyword co-occurrence and clustering

**Figure 3:**
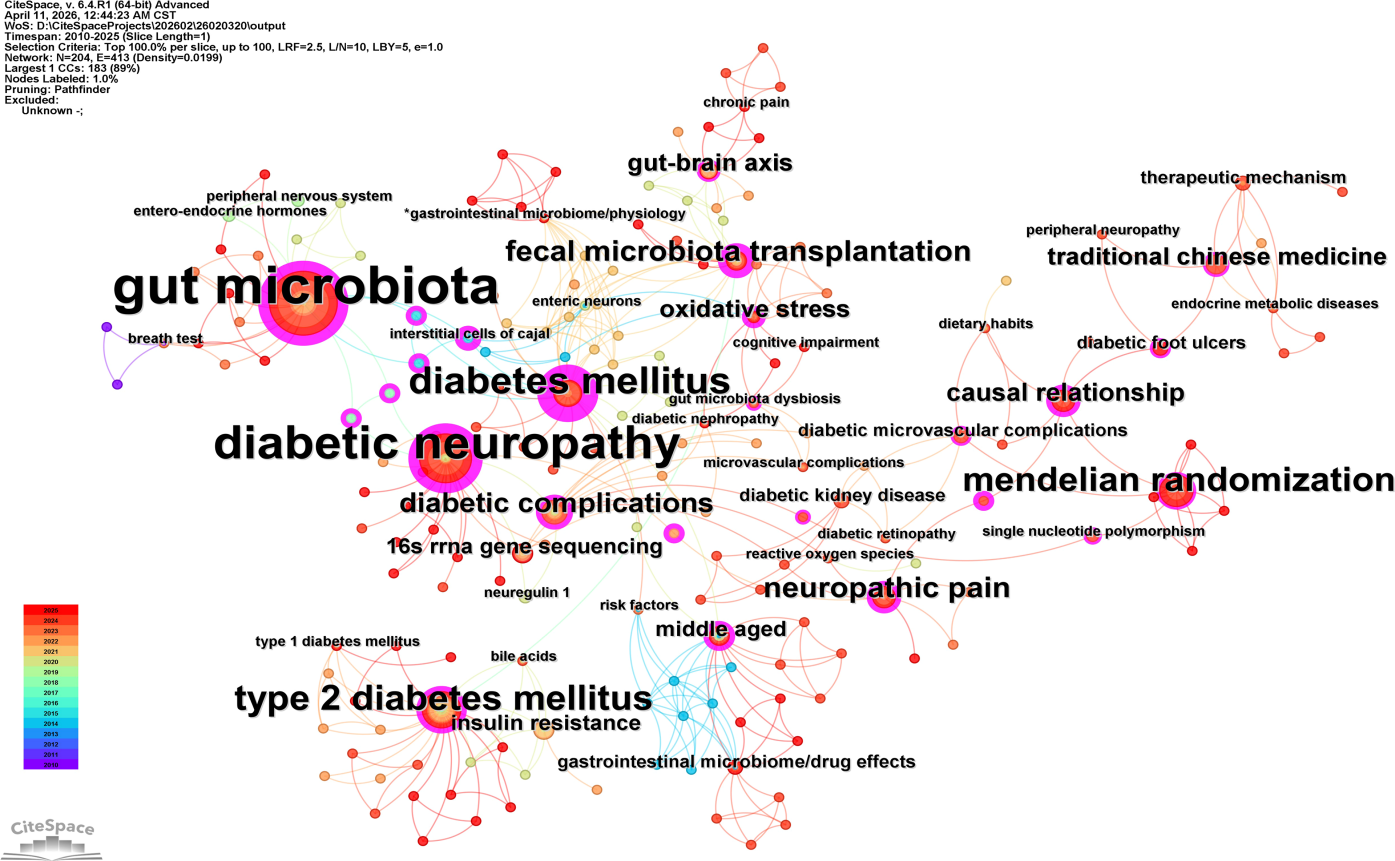
Keyword co-occurrence network of research on gut microbiota and diabetic peripheral neuropathy after synonym merging. The network comprises 204 nodes and 413 links (density = 0.0199). Node size represents frequency of occurrence; lines indicate co-occurrence relationships. Central high-frequency terms include “gut microbiota”, “diabetic peripheral neuropathy”, and “type 2 diabetes mellitus”. Key mechanistic and interventional nodes are color-coded by mean publication year (cool to warm colors denote older to more recent topics).

Following the consolidation of synonyms (e.g., “gut microbiome” and “intestinal flora” were merged into “gut microbiota”; “diabetic neuropathy” was merged into “diabetic peripheral neuropathy”), the keyword co-occurrence network consisted of 204 nodes and 413 links (**Figure 3**), indicating a well-connected research domain (density = 0.0199). Central high-frequency nodes, including “gut microbiota,” “diabetic peripheral neuropathy,” and “type 2 diabetes mellitus,” exhibit dense interconnections with mechanistic themes such as “oxidative stress,” “chronic pain,” and the “gut-brain axis,” as well as interventional strategies like “fecal microbiota transplantation” and “traditional Chinese medicine.”

**Figure 4:**
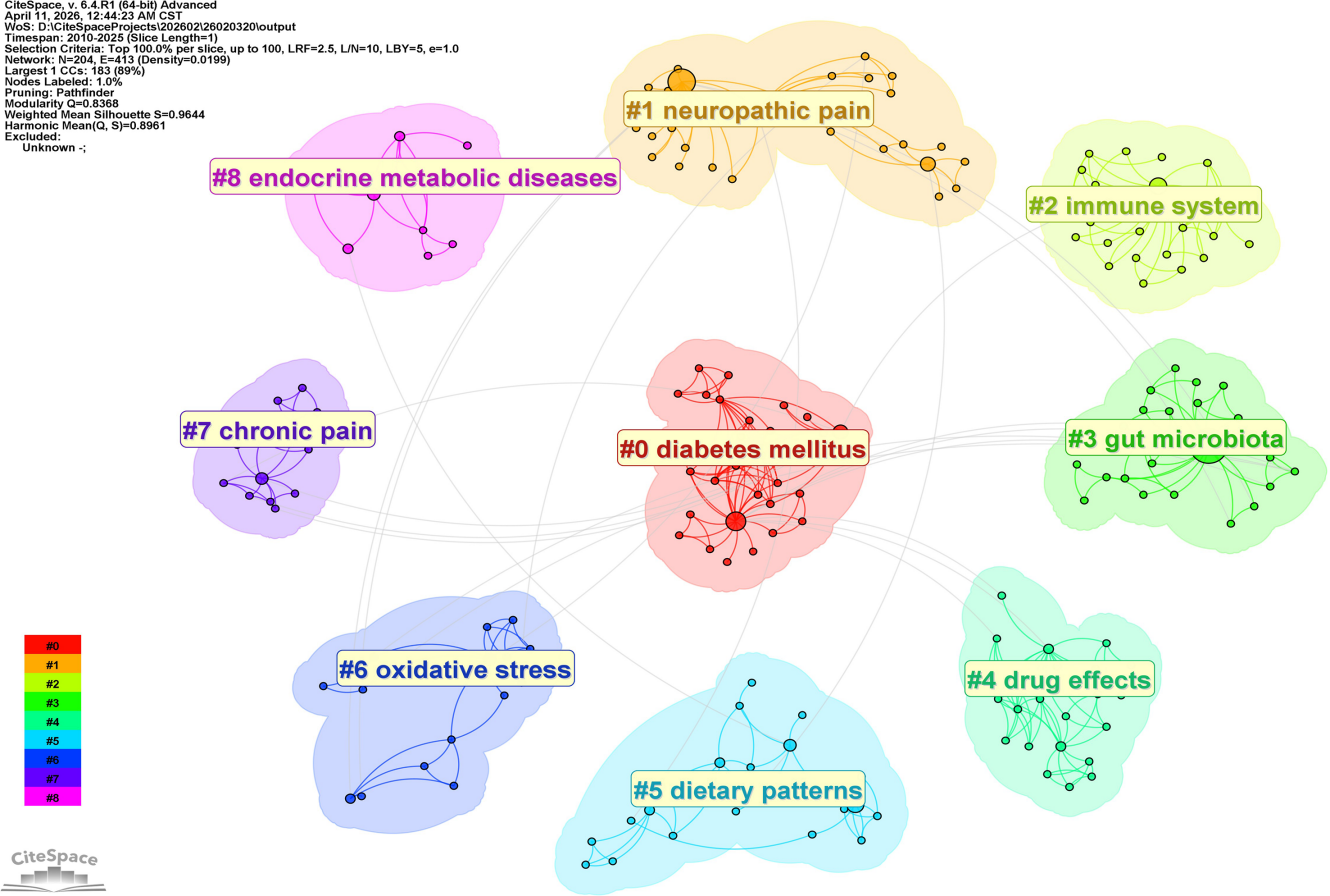
Keyword cluster network of research on gut microbiota and diabetic peripheral neuropathy after synonym merging. The network is partitioned into nine major thematic clusters (Modularity Q = 0.8368, weighted mean Silhouette = 0.9644), each labeled with a title derived from log-likelihood ratio (LLR) tests and distinguished by color. The clusters comprise #0 diabetes mellitus, #1 neuropathic pain, #2 immune system, #3 gut microbiota, #4 drug effects, #5 dietary patterns, #6 oxidative stress, #7 chronic pain, and #8 endocrine metabolic diseases.

Cluster analysis revealed nine major research themes (**Figure 4**), with a Modularity Q value of 0.8368 (Q > 0.3 signifies a significant network structure) and a weighted mean Silhouette score of 0.9644 (S > 0.7 indicates highly credible clustering). These clusters can be conceptually categorized into three layers:

Core Subject ((#0 diabetes mellitus, #3 gut microbiota): These clusters delineate the fundamental scope, centering on the disease context and the microbial community per se (4).
Pathophysiological Mechanisms ((#1 neuropathic pain, #2 immune system, #6 oxidative stress, #7 chronic pain): These clusters represent the principal pathways under investigation. Neuropathic pain constitutes the key clinical manifestation (3). Immune dysregulation and oxidative stress are classical pathological mechanisms that are exacerbated by dysbiosis (11, 12). Chronic pain encompasses the broader pain phenotype (13).
Research Approaches and Modulating Factors ((#4 drug effects, #5 dietary patterns, #8 endocrine metabolic diseases): This layer reflects pharmacological interventions, nutritional modulation, and the broader endocrine context (14). Notably, although “Mendelian randomization” did not form a distinct cluster, it emerged as a high-frequency keyword with a strong citation burst (see Section 3.4) (15, 16). Additionally, “bile acids” persisted as a prominent node in the co-occurrence network, underscoring their emerging mechanistic relevance (5).

### 3.3 Thematic evolution: timeline and timezone views

**Figure 5:**
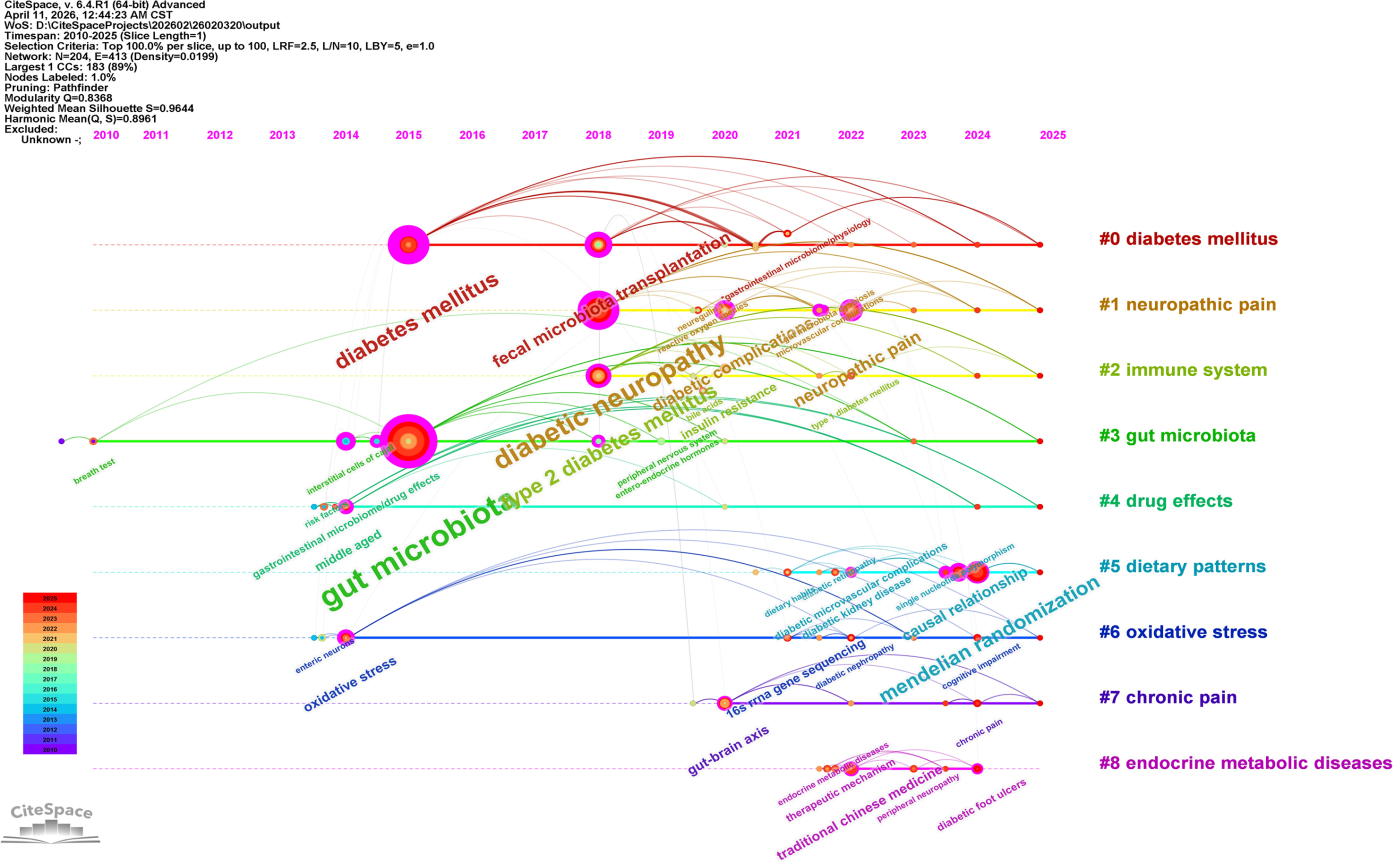
Timeline view of keyword clusters after synonym merging. This visualization depicts the duration and activity level of each thematic cluster over time (Modularity Q = 0.8368, Silhouette = 0.9644). The x-axis represents the temporal span from 2010 to 2025, while the y-axis lists the clusters. Curves connecting keywords within and across clusters illustrate their temporal presence.

**Figure 6:**
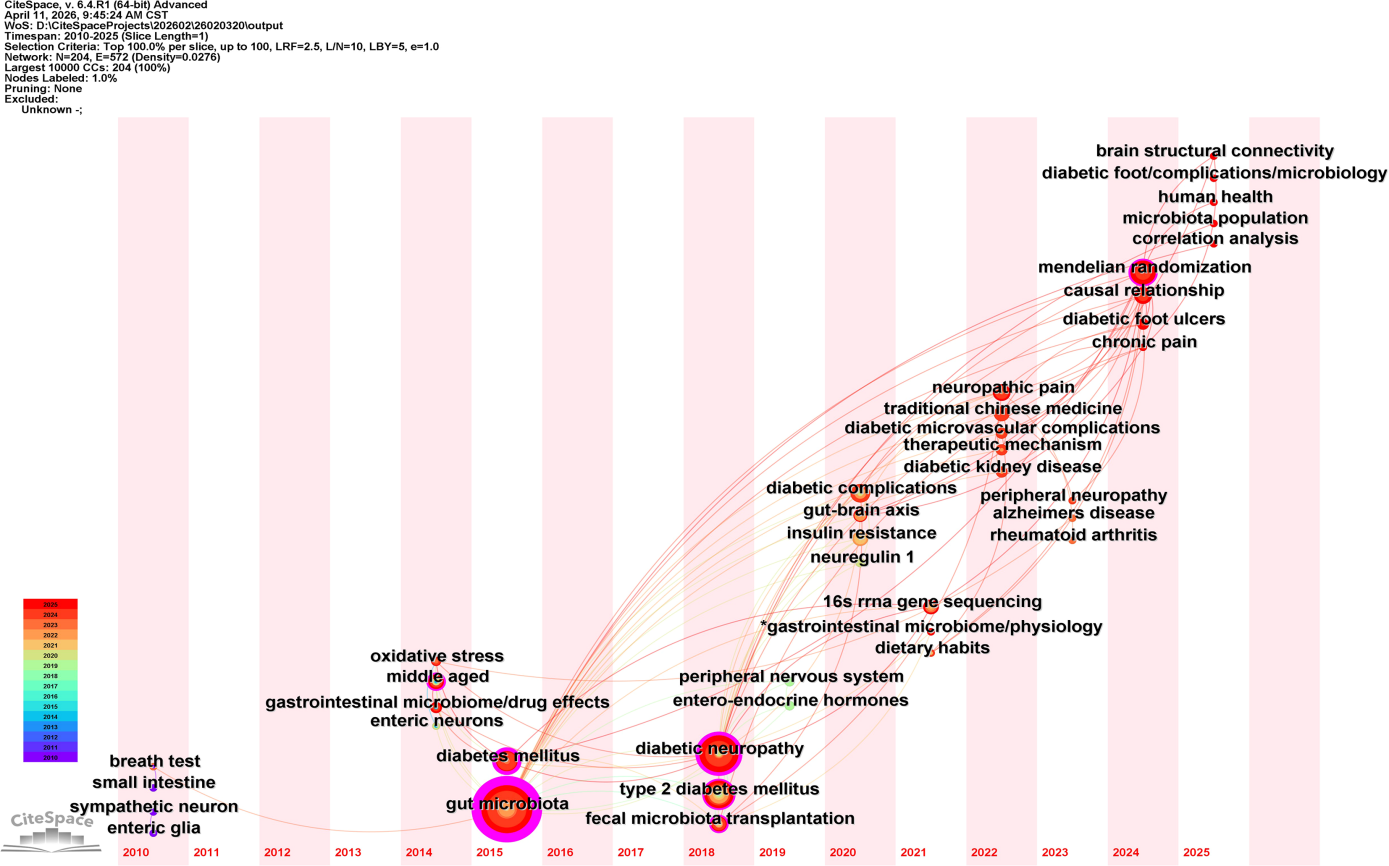
Timezone view of keyword co-occurrence after synonym merging. This visualization displays keywords distributed according to their year of initial prominence (2010–2025). Nodes are positioned in temporal zones based on their publication year, illustrating the conceptual flow and emergence of novel topics over time. Key recent nodes (2024–2025) include “Mendelian randomization”, “causal relationship”, “single nucleotide polymorphism”, and “traditional Chinese medicine”.

The timeline view (**Figure 5**) and timezone view (**Figure 6**) depict the dynamic evolution of research foci. Early-stage research (prior to 2015) primarily focused on fundamental diabetic complications and pathological processes, such as “oxidative stress” and “enteric neurons”. Between 2016 and 2020, keywords including “insulin resistance”, “16S rRNA gene sequencing”, and “peripheral nervous system” gained prominence, reflecting a deeper investigation into mechanistic and methodological foundations. From 2021 onward, the research lexicon shifted markedly toward interventional and analytical approaches, with pronounced bursts observed for “fecal microbiota transplantation”, “gut-brain axis”, and most notably “Mendelian randomization” (2024–2025). Furthermore, “traditional Chinese medicine” and “causal relationship” have emerged as recent frontiers. “Bile acids” and “chronic pain” also appear as notable nodes in the timezone view, indicating sustained scholarly interest in metabolite signaling and pain mechanisms.

### 3.4 Research frontiers: keyword burst detection

**Figure 7:**
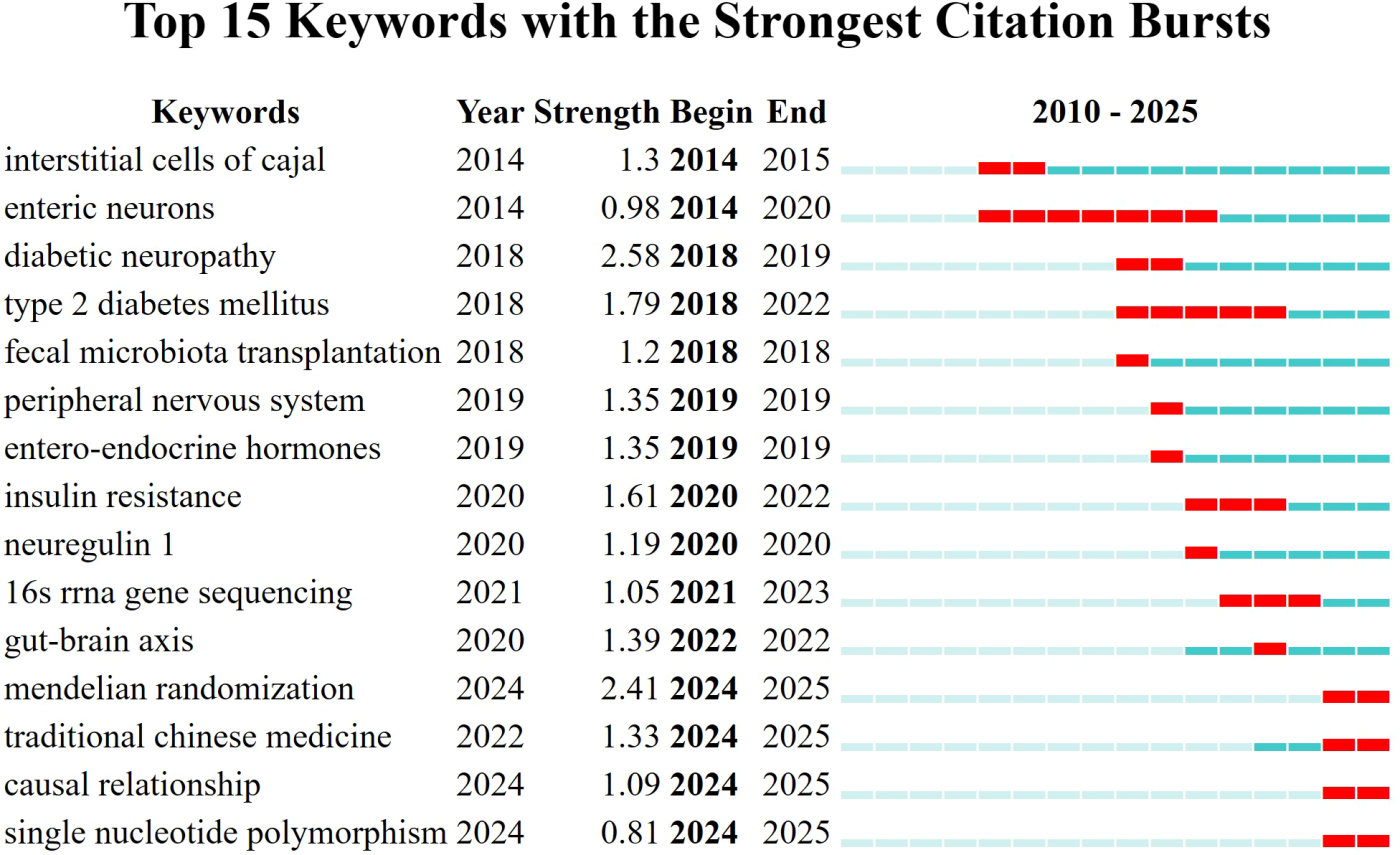
Top 15 keywords with the strongest citation bursts (after synonym merging). Red segments superimposed on the blue timeline indicate the period during which each keyword experienced a citation burst. “Strength” quantifies the intensity of the burst. The strongest burst is observed for “diabetic neuropathy” (Strength = 2.58, 2018–2019), followed by “Mendelian randomization” (Strength = 2.41, 2024–2025) and “type 2 diabetes mellitus” (Strength = 1.79, 2018–2022). Other notable bursts include “fecal microbiota transplantation” (2018), “gut-brain axis” (2022), “traditional Chinese medicine” (2024–2025), and “causal relationship” (2024–2025).

Burst detection analysis (**Figure 7**) quantitatively corroborates this evolutionary trajectory. “Diabetic neuropathy” exhibited the strongest citation burst (Strength = 2.58) during 2018–2019, marking the period when the association was firmly established. “Type 2 diabetes mellitus” also demonstrated a prolonged burst from 2018 to 2022 (Strength = 1.79). Subsequently, “fecal microbiota transplantation” displayed a burst in 2018, and “gut-brain axis” in 2022 (Strength = 1.39). Most notably, “Mendelian randomization” represents the strongest current burst keyword (2024–2025, Strength = 2.41), indicating that the forefront of the field is currently dominated by causal inference studies employing genetic instruments, as exemplified by the works of Li et al. (15) and Tang et al. (17). Other recent bursts encompass “traditional Chinese medicine” (2024–2025, Strength = 1.33) and “causal relationship” (2024–2025, Strength = 1.09). It is noteworthy that “probiotics” did not emerge as a burst keyword following synonym merging, as relevant studies may have been subsumed under broader terms or have not yet attained a citation burst threshold in this dataset.

### 3.5 Intellectual base: document co-citation analysis

**Figure 8:**
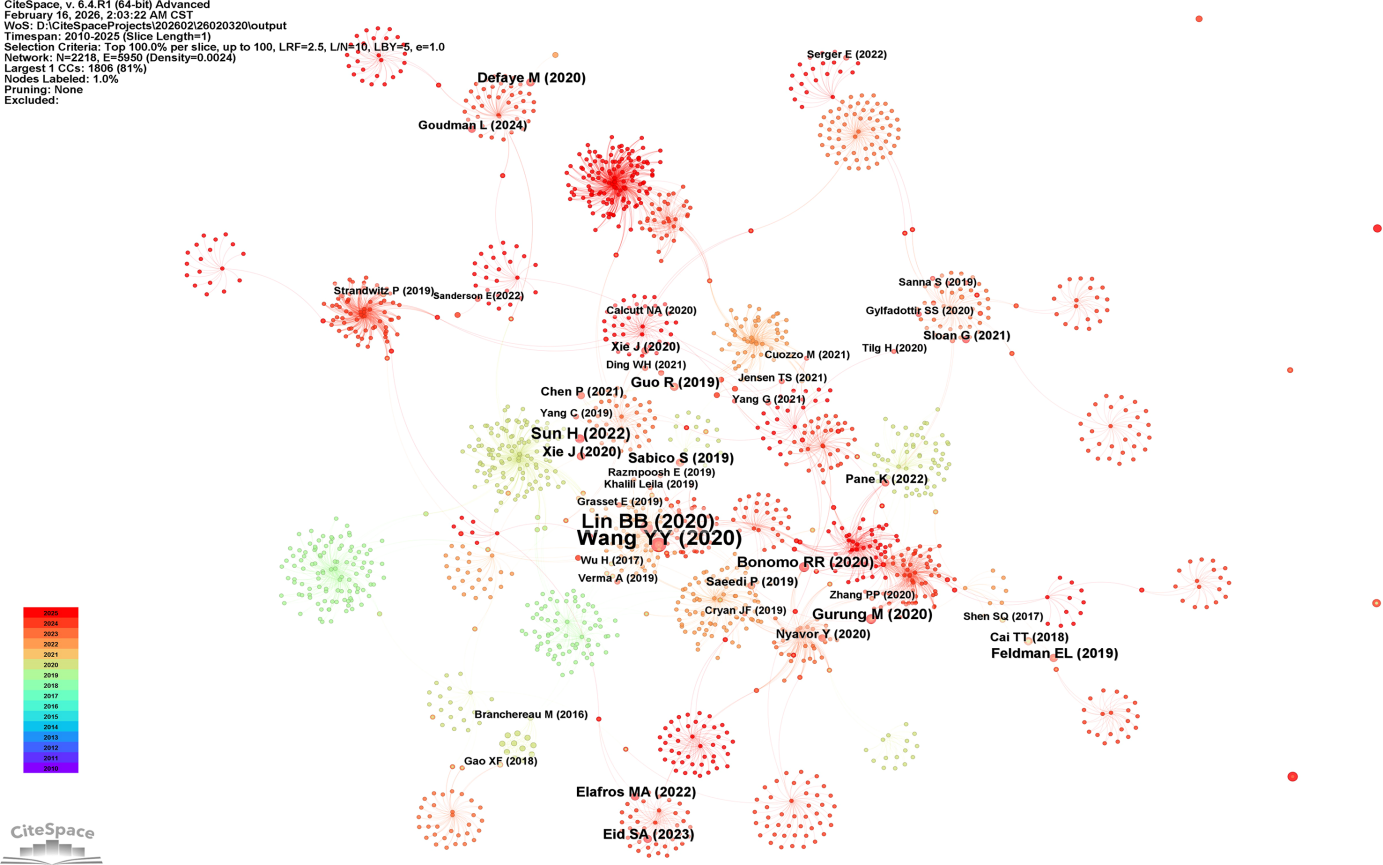
Document co-citation network. Nodes represent cited references; size corresponds to citation frequency. Links denote co-citation relationships. Key foundational papers are labeled.

The document co-citation network (**Figure 8**) consisted of 187 nodes and 342 links, exhibiting a modularity Q value of 0.73 and a mean silhouette score of 0.91. Among the most influential works, the clinical trial conducted by Yang et al. (2023) (9) demonstrated the highest local citation count (n = 24) and a centrality of 0.18, functioning as a central hub. This study provided the first randomized controlled evidence indicating that fecal microbiota transplantation from healthy donors alleviates distal symmetric polyneuropathy independently of glycemic control, proposing the “competing microbial guilds” model. The animal study by Bonomo et al. (2020) (18) exhibited a local citation count of 19 and a centrality of 0.14, identifying butyrate as a key effector molecule that mediates the beneficial effects of FMT on neuropathic pain through immune modulation and mitochondrial function. Other foundational references include the review by Feldman et al. (2019) (1) on diabetic neuropathy (centrality 0.09) and the mechanistic study by Chen et al. (2024) (5) on the TGR5/TRPV1 pathway (centrality 0.07).

## 4 Discussion

This bibliometric analysis offers a comprehensive and dynamic overview of the rapidly evolving research landscape at the intersection of gut microbiota, the gut-brain axis, and diabetic peripheral neuropathy (DPN). The findings confirm and visually delineate the field’s progression along a translational continuum, ranging from observational studies to mechanistic investigations and ultimately to interventional approaches, all integrated within the overarching gut-brain axis framework.

While bibliometric analysis elucidates research patterns, it does not directly validate biological mechanisms or clinical efficacy. Consequently, our interpretations of biological pathways are consistently grounded in the original studies cited, and statements regarding clinical implications are derived from the research trends identified in the literature, rather than from direct evidence synthesis.

Interpretation of publication trend fluctuations: The minor decline in publications in 2024 should not be overinterpreted as indicative of waning interest. Given the search cutoff in January 2025, recent articles from late 2024 may not have been fully indexed in databases such as WoSCC, PubMed, or Scopus. Additionally, the peer-review and publication processes can introduce a lag of several months. The overall trajectory remains one of robust growth.

### 4.1 Overview: the meteoric rise of a translational research field

The observed publication trend directly reflects the paradigm shift in understanding DPN pathogenesis. The stagnation prior to 2018 corresponds to the era dominated by conventional neurocentric and vascular theories. The subsequent explosive growth coincides with the broader acceptance of the “gut-brain axis” in neurobiology and the accumulation of preclinical data linking gut microbiota dysbiosis to neuroinflammation and nerve damage. This field has now solidified as a major frontier in diabetic complications research.

### 4.2 Hotspots and frontiers: deciphering the knowledge structure

#### 4.2.1 From mechanism to modulation: the evolution of research themes

The keyword clusters (**Figure 4**) depict a coherent landscape of the current research ecosystem. The core thematic areas are delineated by #0 diabetes mellitus and #3 gut microbiota, which establish the foundational disease context and the implicated microbial community. Pathophysiological mechanisms are represented by clusters #1 neuropathic pain, #2 immune system, #6 oxidative stress, and #7 chronic pain, highlighting immune dysregulation and oxidative stress as pivotal pathways linking microbial dysbiosis to neuronal injury. Research methodologies and modulating factors are encompassed within #4 drug effects, #5 dietary patterns, and #8 endocrine metabolic diseases, reflecting the pharmacological, nutritional, and broader endocrine-metabolic contexts of investigation.

Although MR did not form an independent cluster, it emerged as a high-frequency keyword exhibiting the strongest citation burst (2024–2025, strength: 2.41), signifying a critical methodological shift towards causal inference. Recent MR analyzes have robustly suggested causal roles for specific bacterial genera, such as Eubacterium and Ruminococcus, in DPN risk, thereby providing a stronger rationale for targeted therapeutic interventions (15, 17). Concurrently, “bile acids” persisted as a prominent node within the co-occurrence network and timezone visualization, underscoring its growing mechanistic significance. The association of bile acids with specific receptor pathways, exemplified by the TGR5/TRPV1 axis, illustrates the field’s progression from characterizing broad dysbiosis to elucidating specific microbiota-derived molecules and their precise neuronal targets (5).

#### 4.2.2 The gut-brain axis: a multi-mechanistic framework

The gut-brain axis provides the overarching conceptual framework that unifies the diverse research themes identified in this analysis. This bidirectional communication network encompasses multiple mechanistic pathways through which the gut microbiota influences peripheral nerve function:

##### 4.2.2.1 Immune-mediated pathway

Gut dysbiosis increases intestinal permeability, facilitating the translocation of LPS and other bacterial products into the systemic circulation. This process triggers TLR4-mediated activation of immune cells, leading to a state of systemic inflammation and neuroinflammation that ultimately damages peripheral nerves (4, 19).

##### 4.2.2.2 Metabolite-mediated pathway

SCFAs, particularly butyrate, function as critical signaling molecules. Research by Bonomo et al. (18) demonstrated that FMT restores butyrate-producing bacteria, which ameliorates neuropathic pain by enhancing mitochondrial function and reducing oxidative stress within dorsal root ganglion neurons.

##### 4.2.2.3 Bile acid-TGR5/TRPV1 pathway

Emerging evidence highlights the role of microbial bile acid metabolism in DPN pathogenesis. Secondary bile acids activate TGR5 on dorsal root ganglion neurons. This activation initiates CREB signaling and subsequent TRPV1 channel activation, leading to nociceptor sensitization and contributing to neuropathic pain (5, 19). This pathway represents a promising therapeutic target, as pharmacological modulation of TGR5 may potentially decouple metabolic dysfunction from neuropathic pain.

#### 4.2.3 Mendelian randomization: establishing causality

The emergence of MR as a keyword exhibiting a strong citation burst (2024–2025) signifies a notable methodological shift toward causal inference within the extant literature. MR employs genetic variants as instrumental variables to infer causal relationships, thereby circumventing limitations inherent in observational studies, such as confounding and reverse causation. Recent MR investigations have identified 14 gut microbiota species with putative causal links to diabetic neuropathy (15). Notably, the Lachnospiraceae family was established as a common risk factor for both diabetic neuropathy and peripheral artery disease (OR = 1.392, 95% CI: 1.031–1.880) (17), whereas Acidaminococcaceae was identified as a shared protective factor (OR = 0.620, 95% CI: 0.460–0.837). These findings furnish genetic evidence supporting the causal involvement of specific microbial taxa in DPN pathogenesis and help prioritize potential therapeutic targets for intervention. However, the identification of causally implicated microbial taxa (e.g., Lachnospiraceae) necessitates validation across diverse ancestral populations, as current genome-wide association studies (GWAS) predominantly comprise individuals of European descent.

### 4.3 Evolutionary trajectory: from association to translation

The temporal evolution revealed by burst and timezone analyzes delineates a coherent research trajectory encompassing four distinct phases:

#### 4.3.1 Phase I (2010–2017):foundation and association

Initial research established the phenomenological association between alterations in GM and DPN. Observational studies reported reduced microbial diversity and depletion of beneficial taxa in DPN patients compared to diabetic controls without neuropathy (14).

#### 4.3.2 Phase II (2018–2021): mechanistic exploration

Subsequent investigations focused on elucidating underlying mechanisms, implicating pathways such as endotoxemia (via LPS), loss of protective microbial metabolites (e.g., SCFAs like butyrate), and intestinal barrier dysfunction. The landmark study by Bonomo et al. (18) (2020) solidified the role of butyrate as a critical effector molecule, redirecting research interest toward postbiotic and metabolite-based therapeutic strategies.

#### 4.3.3 Phase III (2022–2024): causal validation

The current research frontier, prominently featuring “Mendelian randomization,” reflects methodological maturation. Studies are increasingly employing genetic instrumental variables to establish causal relationships and prioritize therapeutic targets with greater confidence.

#### 4.3.4 Phase IV (2023–present): clinical translation

The emergence of “fecal microbiota transplantation (FMT)” and “traditional Chinese medicine” as burst keywords signals active evaluation of GM-targeted interventions. The randomized controlled trial by Yang et al. (9) demonstrated that FMT from healthy donors significantly improved neuropathic symptoms independent of glycemic control, providing the first high-quality clinical evidence for microbiota-directed therapy in DPN.

### 4.4 Foundational studies and their lasting impact

Co-citation analysis delineates seminal studies that have functioned as critical inflection points within the field. The FMT trial conducted by Yang et al. (9) is foundational not only for its clinical outcomes but also for proposing the conceptual model of “competing microbial guilds,” wherein the equilibrium between beneficial butyrate-producing taxa and pro-inflammatory endotoxin-producing taxa determines neurological outcomes. This framework has profoundly influenced subsequent mechanistic research. Similarly, the work by Bonomo et al. (18) consolidated the role of butyrate as a pivotal effector molecule, steering the field toward investigating postbiotic and metabolite-based therapeutic approaches, rather than focusing exclusively on whole-community microbial transplantation.

### 4.5 Clinical implications and future directions

This bibliometric insight delineates several concrete directions for clinical practice and future research:

#### 4.5.1 Biomarker discovery

The emergence of “bile acids” and “Mendelian randomization” as research hotspots indicates heightened scholarly focus on these topics. According to the cited primary studies (20, 21), specific microbial metabolites (e.g., secondary bile acids, formate) have been proposed as candidate biomarkers; however, prospective validation is imperative. Prospective cohorts integrating metagenomics and metabolomics are required to validate these candidate biomarkers.

#### 4.5.2 Therapeutic arsenal expansion

The identification of “fecal microbiota transplantation” as a burst keyword (2018) and “traditional Chinese medicine” (2024-2025) suggests escalating research interest in these interventions. Based on the co-cited clinical trial (9), FMT has demonstrated preliminary efficacy in a single study; nevertheless, future trials are essential to corroborate these findings. The literature also hypothesizes regarding defined consortia or engineered bacteria, but these remain largely experimental. The interaction between microbiota-targeted interventions and first-line pharmacotherapies (e.g., metformin, gabapentinoids) warrants further investigation.

#### 4.5.3 Personalized medicine

The identification of causal microbial taxa through Mendelian randomization (e.g., Lachnospiraceae, Acidaminococcaceae) suggests the potential for microbiome-based patient stratification. Machine learning models that integrate gut metagenomic profiles, genetic variants, and clinical phenotypes may enable the prediction of individual responses to FMT or specific probiotic regimens.

#### 4.5.4 Methodological priorities

Future bibliometric updates should incorporate real-time citation tracking and natural language processing techniques to capture emerging research concepts—such as “postbiotics” and “vagus nerve stimulation”—that are currently underrepresented in the literature.

### 4.6 Future research frontiers

Based on the bibliometric analysis, the following directions are anticipated to emerge as major research frontiers within the next 3–5 years:

#### 4.6.1 Multi-omics integration

Integrating metagenomics, metabolomics, proteomics, and transcriptomics will provide a systems-level understanding of the gut–nerve axis. This holistic approach will facilitate the identification of key regulatory nodes and novel therapeutic targets.

#### 4.6.2 Neural pathway elucidation

The role of the vagus nerve in transmitting microbial signals to the central nervous system and its implications for peripheral neuropathy warrant further investigation. Optogenetic and chemogenetic tools may help delineate these specific neural circuits.

#### 4.6.3 Engineered therapeutics

The development of genetically engineered probiotics capable of producing therapeutic molecules (e.g., butyrate, GLP-1) *in situ* represents a next-generation strategy for microbiota-based therapy.

#### 4.6.4 Precision microbiome medicine

Stratifying DPN patients according to gut microbiome profiles will support personalized treatment strategies. Machine learning algorithms trained on multi-omics data may predict individual responses to FMT or specific probiotic formulations.

#### 4.6.5 Long-term safety and efficacy

Large-scale, multi-center randomized controlled trials with extended follow-up periods are required to establish the long-term safety and efficacy of microbiota-based interventions for DPN.

## 5 Strengths and limitations

Compared with traditional narrative reviews, this study employs statistical bibliometric techniques to analyze 133 publications, offering a more systematic and comprehensive overview of research hotspots and developmental trends in this field from 2010 to the present. The use of bibliometric tools strengthens the methodological rigor of our findings. The results provide valuable insights for researchers and clinicians navigating the rapidly evolving landscape of GM–DPN research.

Nevertheless, several limitations should be noted. First, although the integration of three complementary databases—WoSCC, PubMed, and Scopus—represents a widely used and authoritative approach, their coverage remains confined to a specific scope within the field. Moreover, this analysis included only English-language publications, potentially omitting significant studies published in other languages, particularly Chinese research that may contain relevant findings. Second, CiteSpace analysis depends on keyword selection, which can vary across researchers. This subjectivity may influence the analytical outcomes and lead to the omission of certain important research themes. Third, the rapid evolution of the field implies that the most recent trends may not be fully captured in our analysis. Finally, bibliometric analysis reflects research output and citation dynamics rather than direct measures of research quality; high-frequency keywords do not necessarily correspond to the most critical scientific questions.

## 6 Conclusion

This investigation represents the inaugural bibliometric analysis of the gut microbiota–diabetic peripheral neuropathy research domain, systematically delineating its developmental trajectory and intellectual evolution. From 2010 onward, scholarly output demonstrated a gradual increase until 2017, followed by a pronounced acceleration post-2018, with peak publication volumes recorded in 2023 (36 articles) and 2024 (30 articles). Keyword co-occurrence and cluster analysis identified nine principal thematic clusters, categorized into core subjects (diabetes mellitus, gut microbiota), pathophysiological mechanisms (neuropathic pain, immune system, oxidative stress, chronic pain), and methodological approaches (drug effects, dietary patterns, endocrine metabolic diseases). Burst detection analysis revealed that “Mendelian randomization” (2024–2025, strength 2.41) and “traditional Chinese medicine” (2024–2025, strength 1.33) constitute the most emergent research frontiers, whereas “fecal microbiota transplantation” exhibited an earlier burst phase (2018). Co-citation network analysis underscored the clinical trial by Yang et al. (local citation count 24, centrality 0.18) and the animal study by Bonomo et al. (local citation count 19, centrality 0.14) as seminal contributions.

These findings suggest that the field is undergoing a transition from descriptive associative studies toward causal inference methodologies (e.g., Mendelian randomization) and translational therapeutic interventions (e.g., fecal microbiota transplantation, traditional Chinese medicine). The gut-brain axis persists as the predominant conceptual framework organizing current research. Future research directions, as extrapolated from the extant literature, encompass large-scale clinical corroboration of microbiota-based therapies, integrative multi-omics approaches, and personalized microbiome-based therapeutics. Nevertheless, it is imperative to acknowledge that bibliometric trends reflect scholarly activity rather than validated biological mechanisms or clinical efficacy.

## Data Availability

The original contributions presented in the study are included in the article/supplementary material. Further inquiries can be directed to the corresponding authors.
